# Diabetes potentiates the emergence and expansion of antibiotic resistance

**DOI:** 10.1126/sciadv.ads1591

**Published:** 2025-02-12

**Authors:** John C. Shook, Christopher J. Genito, Benjamin P. Darwitz, Kaleb J. Tyson, Amanda Z. Velez, Sophia K. Bridwell, Joshua B. Parsons, Sarah E. Rowe, Christopher W. Marshall, Brian P. Conlon, Lance R. Thurlow

**Affiliations:** ^1^ Department of Microbiology and Immunology, University of North Carolina at Chapel Hill, Chapel Hill, NC 27599, USA.; ^2^ Department of Biomedical Sciences, Adams School of Dentistry, University of North Carolina at Chapel Hill, Chapel Hill, NC 27599, USA.; ^3^ Department of Biological Sciences, Marquette University, Milwaukee, WI 53233, USA.; ^4^ Division of Infectious Diseases, Duke University School of Medicine, Durham, NC 27710, USA.; ^5^ Marsico Lung Institute, University of North Carolina at Chapel Hill, Chapel Hill, NC 27599, USA.

## Abstract

Individuals with diabetes mellitus frequently develop severe skin and soft tissue infections (SSTIs) that are recalcitrant to antibiotic treatment. We examined how diabetes affects the emergence of antibiotic resistance in a *Staphylococcus aureus* SSTI. We determined that *S. aureus* evolves antibiotic resistance rapidly in diabetic mice, while resistance did not occur in nondiabetic mice over the course of infection. Diabetes-associated immune cell dysfunction plays a minor role in the emergence of resistance, while hyperglycemia plays a dominant role facilitating the expansion and takeover of resistant mutants in diabetic infections. Furthermore, vancomycin intermediate resistant isolates display a pronounced fitness defect in nondiabetic mice but not in diabetic mice. Together, these data suggest that the diabetic infection environment represents an ideal reservoir for the emergence and proliferation of antibiotic resistance. Controlling the blood sugar of diabetic mice with insulin resulted in significantly decreased incidence of antibiotic-resistant *S. aureus*.

## Teaser

Diabetic infections are a reservoir for the emergence and proliferation of antibiotic resistance in *Staphylococcus aureus*.

## INTRODUCTION

There are nearly 530 million people worldwide with diabetes mellitus (DM), and that number is projected to be 1.3 billion people by the year 2050 (*1*). In addition, it is estimated that 44.7% of individuals worldwide with DM are unaware of their DM status (*2*). Aberrant insulin signaling is the primary cause of hyperglycemia. The two major types of diabetes are type 1 (T1DM, insulin deficiency) and type 2 (T2DM, insulin resistance) with individuals with T2DM frequently requiring treatment with insulin. People with DM are more susceptible to bacterial infection, leading to a high frequency of severe and chronic infections in individuals with DM (*3*–*12*). Skin and soft tissue infections (SSTIs) are the most common infections in individuals with diabetes (*13*). Uncontrolled diabetes results in hyperglycemia, leading to elevated blood and tissue glucose concentrations that increase the risk for SSTIs. SSTIs in diabetic individuals are frequently severe and often necessitate amputation especially in the extremities such as toes and feet (*3*, *14*–*17*).

The diabetic infection microenvironment is complex with several factors that contribute to infection frequency and severity as well as antibiotic treatment failure. The increased susceptibility to infection in individuals with diabetes is due to a combination of factors including immunosuppression, hyperglycemia, and lack of vascularization in the extremities (*4*, *7*, *8*, *11*, *13*, *14*, *18*–*22*). Diabetes-associated immune suppression results in dysfunctional phagocytes that have reduced oxidative burst resulting in deficiencies in bacterial clearance (*14*, *23*). Hyperglycemia can greatly enhance the progress of bacterial infection and bacterial virulence genes have been shown to be up-regulated in mouse infection models of hyperglycemia (*24*). *Staphylococcus aureus* is the most prevalent pathogen associated with diabetic SSTIs (*20*–*25*). *S. aureus* uses glucose as a preferential carbon source and becomes hypervirulent in a diabetic SSTI infection (*14*). In addition, nonobese diabetic mice have a delayed immune response and impaired phagocytic killing of *S. aureus* in blood and tissues (*25*, *26*). As a consequence of more frequent and severe infections in patients with DM, antibiotic intervention is more prevalent as well as higher rates of antibiotic treatment failure (*27*, *28*).

Antimicrobial resistance (AMR) further complicates the treatment of infection in individuals with DM. AMR is an emerging threat to global public health and was responsible for more than 4 million deaths in 2019, with projections reaching 10 million deaths by the year 2050 (*29*). In addition, the economic impact of AMR is estimated to surpass US$1 trillion in health care costs by 2050 (*30*). The diabetic microenvironment supports more invasive and severe infections, which results in an increased usage of antibiotics in an attempt to control these infections. Concerningly, patients with DM have seen a 60% increase in antibiotic prescriptions for lower respiratory infections (*31*). It has recently been shown that the evolution of antibiotic resistance occurs more readily in an immunocompromised host (*32*). The diabetic SSTI is both hyperglycemic and immunocompromised; therefore, we sought to determine whether *S. aureus* would more rapidly evolve antibiotic resistance in a diabetic infection environment compared to a nondiabetic infection.

In the studies presented here, we show that rifampicin-resistant (Rif^R^) mutants only emerge from SSTIs in diabetic mice and are never observed in healthy mice. Once present, rifampicin-resistant mutants rapidly take over the bacterial population under antibiotic pressure in as little as 5 days after infection. We find that immune suppression contributes to the emergence resistance, but the increased proliferation is primarily a consequence of increased glucose availability, facilitating the rapid growth of resistant bacteria. These findings demonstrate that the infection environment in diabetes is an ideal reservoir for emergence and proliferation of AMR *S. aureus*. The rapid growth of the diabetic population combined with the concurrent rise of AMR is a serious global health concern (*1*, *29*). Our work provides a basic understanding of how *S. aureus* evolves AMR in diabetic SSTIs and how the diabetic infection microenvironment facilitates the rapid expansion of these resistant bacteria. In total, this work establishes a direct relationship between diabetes and the emergence and proliferation of AMR. The data presented here may inform the development of treatment strategies and highlight the crucial need for the development and implementation of more effective antibacterial compounds to improve infection outcomes in individuals with diabetes.

## RESULTS

### Significant emergence of an antibiotic-resistant bacterial population in an animal model of diabetes

**Fig. 1.**
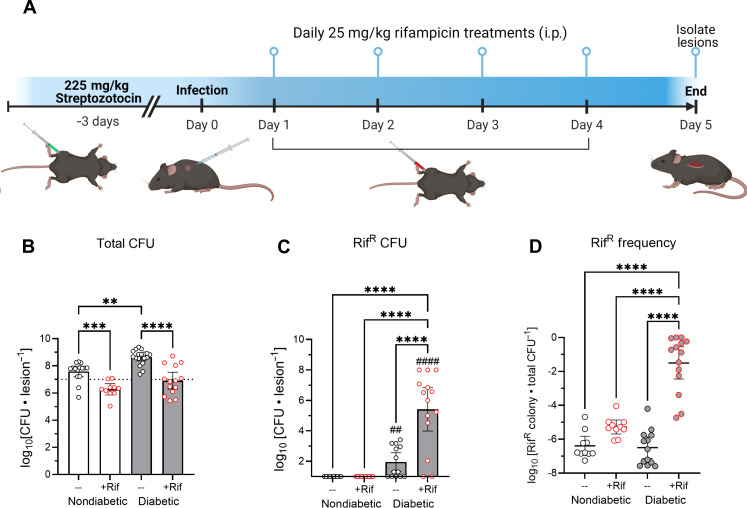
Emergence of antibiotic resistance during rifampicin (Rif) treatment in diabetic mice. (**A**) Schematic of the diabetic skin infection model with rifampicin treatment. Diabetes was induced in mice using streptozotocin 3 days before subcutaneous inoculation with *S. aureus.* Mice were treated daily with rifampicin on days 1 to 4. Lesions were isolated from animals on day 5, and CFUs were enumerated from tissues. (**B**) Total *S. aureus* CFUs recovered from lesions on day 5. (**C**) Rifampicin-resistant *S. aureus* CFUs recovered from lesions on day 5. (**D**) Frequency of rifampicin-resistant colonies (Rif^R^) relative to the total recovered CFUs; note that multiple frequencies were calculated at the limit of detection, and thus, the resulting data represent the maximum possible Rif^R^ frequency. The dotted line indicates the 10^7^ CFU inoculum*.* Data are shown as geometric mean with 95% CI. **P < 0.01, ***P < 0.001, ****P < 0.0001, ANOVA with Tukey’s correction for multiple comparisons. ^##^*P* < 0.01, ^####^*P* < 0.0001, one-sample *t* test to determine whether geometric mean is significantly above the limit of detection. i.p., intraperitoneal.

The prevalence of people with diabetes is increasing yearly (*1*, *2*). In addition, people with diabetes have been shown to have increased incidence of AMR infections (*33*). Therefore, we sought to determine whether the diabetic infection microenvironment contributes to the emergence of AMR. We induced diabetes in wild-type C57BL/6J mice by streptozotocin (STZ) injection, followed by subcutaneous injection of 10^7^ colony-forming units (CFUs) of a methicillin-resistant USA300 *S. aureus* strain, JE2 (Fig. 1A). To model the emergence of antibiotic resistance, we treated infected mice with systemic administration of rifampicin. Rifampicin resistance readily occurs in *S. aureus* at a high frequency due to several single–base pair changes in the gene *rpoB*, which encodes the β subunit of RNA polymerase (*34*). Rifampicin was administered for four consecutive days following *S. aureus* inoculation, then bacterial burdens were enumerated from lesions 5 days after infection. We found this regimen to be effective for treating murine models of *S. aureus* SSTI over a 4-day treatment period (Fig. 1B). Consistent with previous findings, bacterial burdens were significantly higher in diabetic mice than in nondiabetic mice (*14*). Rifampicin treatment reduced bacterial burdens in diabetic and nondiabetic mice by ~2 logs, showing that the antibiotic was still effective in the context of a diabetic infection. However, the difference in rifampicin-resistant (Rif^R^) *S. aureus* CFUs recovered from the lesions after rifampicin treatment between diabetic and nondiabetic animals was notable, with a high frequency of rifampicin resistance observed in the lesions of diabetic mice (Fig. 1, C and D). More than 10^5^ CFUs of Rif^R^ *S. aureus* were recovered from the lesions of diabetic mice following antibiotic treatment, while no Rif^R^ *S. aureus* CFUs were recovered from the lesions of nondiabetic mice (Fig. 1C). In multiple rifampicin-treated diabetic mice, Rif^R^ *S. aureus* emerged as the dominant bacterial population compared to the rifampicin-sensitive population (Fig. 1D). Minimal inhibitory concentration (MIC) assays were performed on three of the colonies from the rifampicin-containing plates and the MIC was 256 μg/ml, a 32,000-fold increase from the parent strain. These observations suggest that the diabetic infection microenvironment supports the emergence of rifampicin resistance, especially as resistance was never observed in nondiabetic mice. The fact that a subset of diabetic infections showed a near 100% rifampicin-resistant population over a 5-day period strongly suggested that the diabetic environment allows the Rif^R^ *S. aureus* population to rapidly expand under antibiotic pressure.

### Rifampicin resistance is caused by high-frequency mutations in the *rpoB* gene

**Fig. 2.**
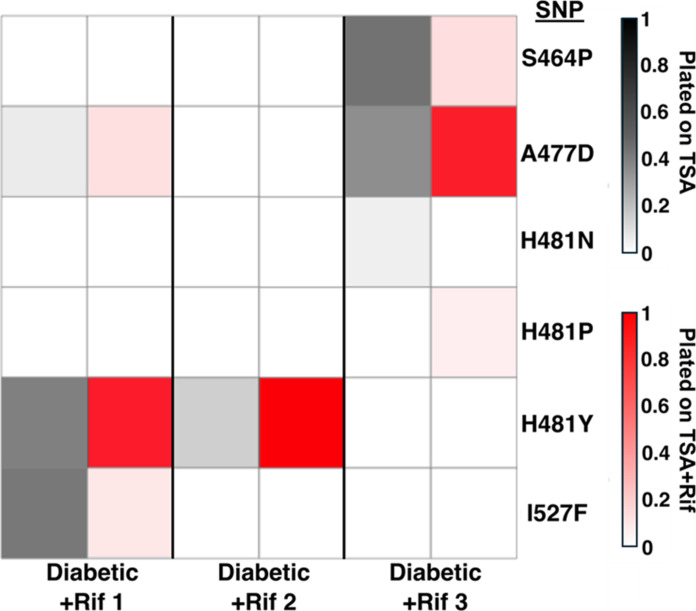
Allele frequencies of the different SNPs in *rpoB* in the rifampicin-treated diabetic mice (*n* = 3 biological replicates). The columns indicate replicate populations and whether they were plated on tryptic soy agar (TSA) (grayscale) or TSA plates supplemented with rifampicin (+Rif) (red color scale).

We used whole population genome sequencing to determine what is driving the emergence of Rif^R^ in the diabetic infection microenvironment. We identified 62 total mutations across all treatments, with two nondiabetic, untreated populations having no mutations detected when compared with their ancestor (fig. S1). Because rifampicin interacts with the β subunit of RNA polymerase, which is encoded by the *rpoB* gene, resistance to rifampicin is typically acquired through mutations in the *rpoB* gene (*34*). In response to rifampicin exposure, multiple nonsynonymous single nucleotide polymorphisms (SNPs) were identified in *rpoB* in diabetic mice (Fig. 2). We observed up to three different *rpoB* mutations in the same population and the mutations H481Y or A477D were enriched on the rifampicin-selective plates, possibly indicating a trade-off between SNPs that confer higher resistance versus those that were more fit in the mouse (Fig. 2).

We hypothesized that the reason for the higher incidence of Rif^R^ colonies and the high frequency of *rpoB* mutations in the diabetic +Rif lesions was the larger population sizes of *S. aureus* observed in the diabetic mice (Fig. 1D). These larger population sizes in diabetic lesions mean a larger mutation supply for selection to act upon. To this end, we estimated the minimum possible number of mutations given the starting and ending population sizes and a constant mutation rate of 0.00035 mutations per cell division (fig. S2). According to our estimates, diabetic mice had a greater number of possible mutations (>10^5^) and we found no evidence for hypermutators in any condition (*35*, *36*). Therefore, the rapid rise in resistance to rifampicin is most likely due to the greater number of replicating cells in the diabetic mice, allowing for more mutations to be sampled.

### Immune suppression has a minor contribution to the emergence of rifampicin-resistant *S. aureus*

**Fig. 3.**
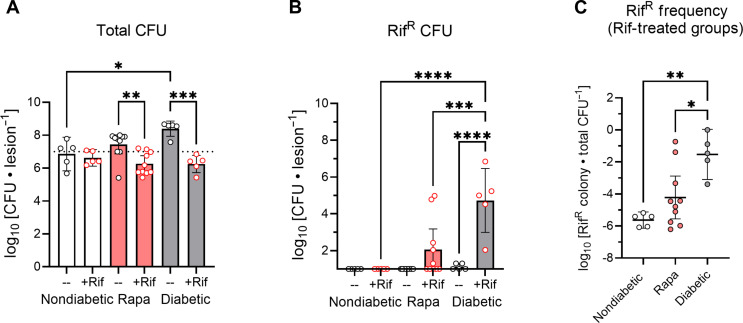
Emergence of rifampicin resistance during host innate immune suppression. Mice were treated with rapamycin (Rapa) to inhibit host innate immune cell bactericidal free radical production. Mice were subcutaneously inoculated with 10^7^ CFU JE2, the lesions were harvested 5 days after infection, and CFUs were enumerated. (**A**) Total *S. aureus* CFUs, and (**B**) *S. aureus* Rif^R^ CFUs. (**C**) Frequency of rifampicin-resistant colonies (Rif^R^) relative to the total recovered CFUs from rifampicin-treated mice; note that multiple frequencies were calculated at the limit of detection; thus, the resulting data represent the maximum possible Rif^R^ frequency. Data are shown as geometric mean with 95% CI. *P < 0.05, **P < 0.01, ***P < 0.001, ****P < 0.0001, ns = not significant, ANOVA with Tukey’s correction for multiple comparisons.

Because we did not observe any genomic signatures that resulted in increased mutation rates in the diabetic infection microenvironment, we wanted to determine whether there were bacterial or host factors that contributed to the increased emergence of Rif^R^ *S. aureus*. It has been well documented that individuals with DM experience a degree of immunosuppression caused by impaired insulin signaling (*4*, *37*). Given the recent work suggesting that the evolution of antibiotic resistance occurs more readily in immunocompromised hosts (*32*), we examined whether the increased incidence of Rif^R^ *S. aureus* in a diabetic host is due to increased bacterial growth resulting from immunosuppression. *S. aureus* infection is exacerbated in a diabetic host due, in part, to inhibited bactericidal free radical production by phagocytes, owing to impaired glucose transporter GLUT1 signaling upon immune cell activation (*14*). We recently showed that rapamycin treatment of nondiabetic mice similarly exacerbates *S. aureus* SSTIs by impairing phagocyte GLUT1 signaling and free radical production (*23*). To simulate diabetic immune suppression in the absence of hyperglycemia, we examined the AMR emergence potential of *S. aureus* in nondiabetic mice treated with rapamycin. Although blood glucose levels were slightly increased in rapamycin-treated mice on the day of infection, their blood sugar was similar to nondiabetic mice by day 5 (fig. S3, B and C). *S. aureus* lesion burden trended higher in rapamycin-treated mice than control mice (Fig. 3A). Rifampicin treatment was effective in significantly reducing bacterial burden in rapamycin-treated mice, with no significant differences in total *S. aureus* CFUs recovered from the lesions of rifampicin-treated groups. We detected Rif^R^ CFUs within 50% of the lesions from rapamycin-treated mice, compared to 0% of lesions from control nondiabetic mice and 100% of lesions from diabetic mice (Fig. 3B). There was a trend in increased Rif^R^ frequency in rapamycin-treated mice, but it was not significant, which could be explained by the slightly elevated blood sugar levels observed on infection day (Fig. 3C and fig. S3B). We therefore concluded that immune suppression may contribute to the emergence of rifampicin resistance, but it is not responsible for the rapid expansion of Rif^R^ *S. aureus* mutants during diabetic infection. It should be noted that *S. aureus* inoculums used for infection in these experiments were confirmed to have undetectable Rif^R^ CFUs per mouse (<1 CFU), and thus, Rif^R^ CFUs recovered from the lesions likely represent de novo mutations during the course of infection. This may also explain why almost no Rif^R^ CFUs were recovered from diabetic mice in the absence of antibiotic pressure (Fig. 3B), which provided further evidence that diabetes does not confer a hypermutable environment.

### *S. aureus* glucose uptake and *acnA* activity play a role in the expansion of Rif^R^ in diabetic hosts

Because immune suppression alone did not seem to account for the high incidence of Rif^R^ in diabetic mice (Fig. 3), we wanted to assess the contribution of elevated host glucose and *S. aureus* metabolism on the expansion of Rif^R^ *S. aureus*. To determine whether glucose contributed to Rif^R^ emergence, we assessed the effect of glucose on the expansion of Rif^R^ *S. aureus* in vitro (fig. S4, A to C)*.* Once grown to stationary phase in the absence of glucose, a background level of ~10^2^ CFUs of Rif^R^ *S. aureus* were present, which is consistent with a high level of spontaneous mutation (*38*). We then supplemented the cultures with 15 mM glucose [tryptic soy broth (TSB) +Glc] or a carbon equivalent amount of casamino acids (TSB +aa). We found that, under antibiotic pressure [rifampicin (10 μg/ml)] over a 48-hour time frame, Rif^R^ *S. aureus* were only able to expand when glucose was present (fig. S4). From these data, we concluded that the emergence of Rif^R^ *S. aureus* is contingent on the presence of glucose in vitro.

*S. aureus* uses distinct mechanisms to rapidly ingest and metabolize glucose in its environment. *S. aureus* encodes four glucose phosphotransferase system (PTS) transporters that drive the uptake and phosphorylation of glucose (*39*). In glucose-replete conditions, *S. aureus* rapidly performs glycolytic metabolism to generate pyruvate in concentrations that exceed the flux of the tricarboxylic acid (TCA) cycle (*40*–*42*). To capitalize on excess pyruvate generation, *S. aureus* can simultaneously flux pyruvate through TCA cycle and fermentative pathways in a redox-balanced manner to supplement its growth (fig. S4A) (*43*). As such, we investigated whether the expansion of Rif^R^ in *S. aureus* is driven by its relatively expanded repertoire of glucose PTS transporters and whether it requires aconitase, the first enzyme in the TCA cycle. To accomplish this, we used a glucose PTS-knockout *S. aureus* mutant (ΔG4) and a mutant *S. aureus* strain with diminished TCA cycle activity (*acnA*::Tn), respectively. We did not observe the expansion of Rif^R^ in either the ΔG4 mutant or the *acnA*::Tn mutant in our in vitro system (fig. S4, D and E). This suggested to us that both glucose uptake and TCA cycle activity were important to the expansion of *S. aureus* Rif^R^ mutants.

**Fig. 4.**
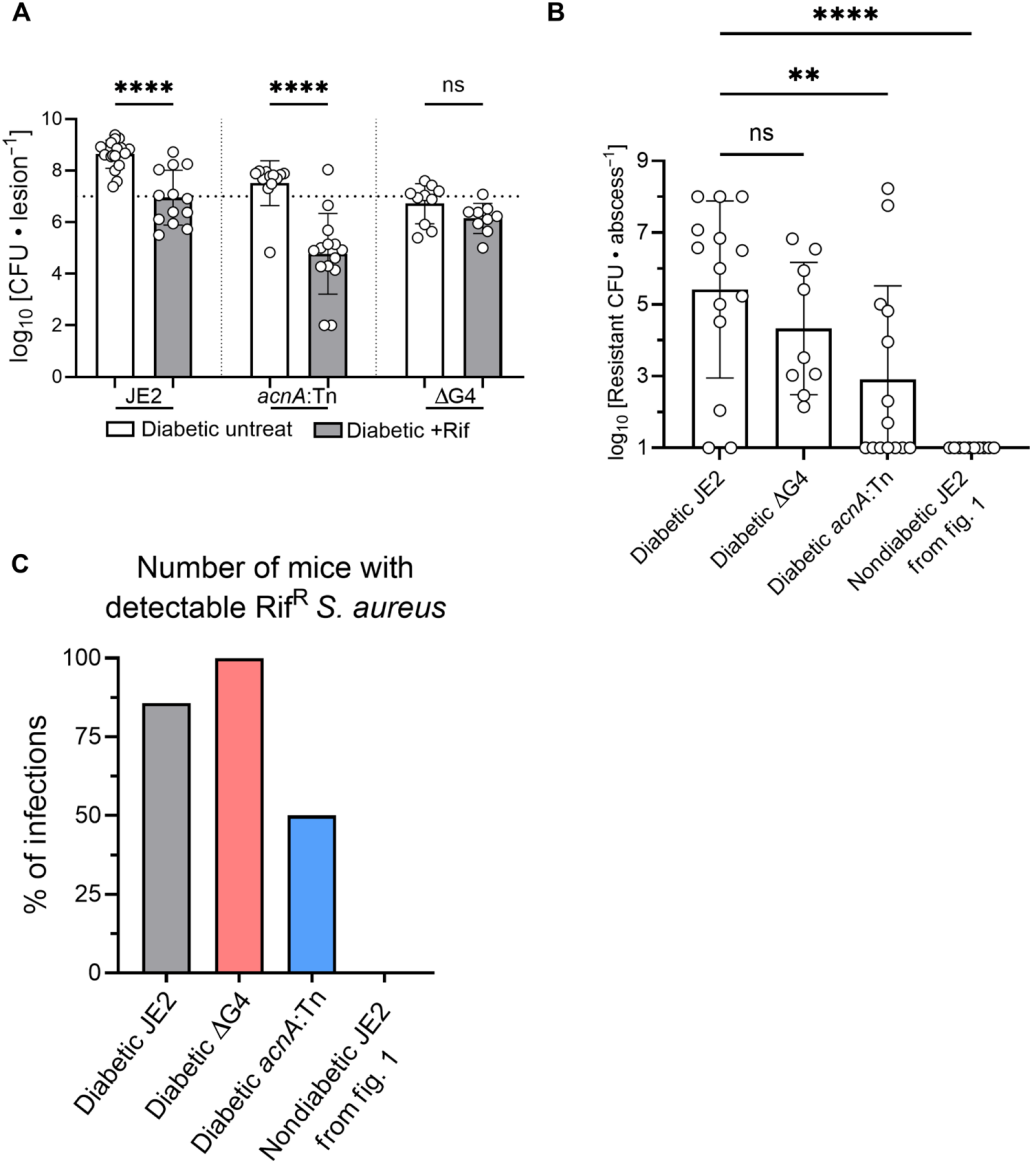
TCA cycle activity contributes to the emergence of rifampicin resistance in *S. aureus* during diabetic infection. (**A**) Diabetic mice were infected with *acnA*::Tn or ∆G4 *S. aureus*. Data from diabetic mice infected with WT *S. aureus* are from Fig. 1, for reference. Mice were treated with rifampicin, and bacterial burdens from lesions were enumerated. (**B**) Rifampicin-treated samples (+Rif) were enumerated on plates containing rifampicin (1 μg/ml). Nondiabetic data from Fig. 1 were used for comparison. (**C**) The number of mice with detectable rifampicin-resistant CFUs was enumerated. Data are shown as the SD from the mean. **P < 0.01, ****P < 0.0001, ns = not significant, ANOVA with Tukey’s correction for multiple comparisons.

While our in vitro data suggested an importance for glucose uptake and TCA cycle activity in the expansion and potential emergence of Rif^R^ *S. aureus* mutants (fig. S4, D and E), testing of these *S. aureus* mutants in our in vivo SSTI model revealed more nuanced results (Fig. 4). Rifampicin treatment of JE2 and *acnA*::Tn infections resulted in a significant reduction of the bacterial burdens (Fig. 4A). The ∆G4 mutant did not display a reduction in bacterial burden following rifampicin treatment. However, when Rif^R^ *S. aureus* from these infections was enumerated, we observed a small, but not statistically significant, decrease in the mean burden of Rif^R^ *S. aureus* from the ∆G4 background compared to WT, and a significant decrease in the amount of Rif^R^ CFUs in the *acnA*::Tn infection (Fig. 4B). We suspected that the low burden of *acnA*::Tn *S. aureus* likely contributed to the lower burden of Rif^R^ CFUs recovered from the *acnA*::Tn infection. While we detected Rif^R^ CFUs in all of the mice infected with ∆G4 *S. aureus*, only 50% of the mice infected with the *acnA*::Tn mutant had detectable levels of Rif^R^ *S. aureus* (Fig. 4C), suggesting that *acnA* activity may be playing a role in the emergence of Rif^R^ *S. aureus* during infection. These in vivo results suggested that maximal glucose uptake by *S. aureus* may play a minor role in the expansion, but not the emergence, of Rif^R^ mutants, while aconitase activity and potentially the flux of pyruvate through the TCA cycle seemed to play an important role in Rif^R^ emergence.

### The diabetic environment supports the proliferation of rifampicin-resistant *S. aureus*

**Fig. 5.**
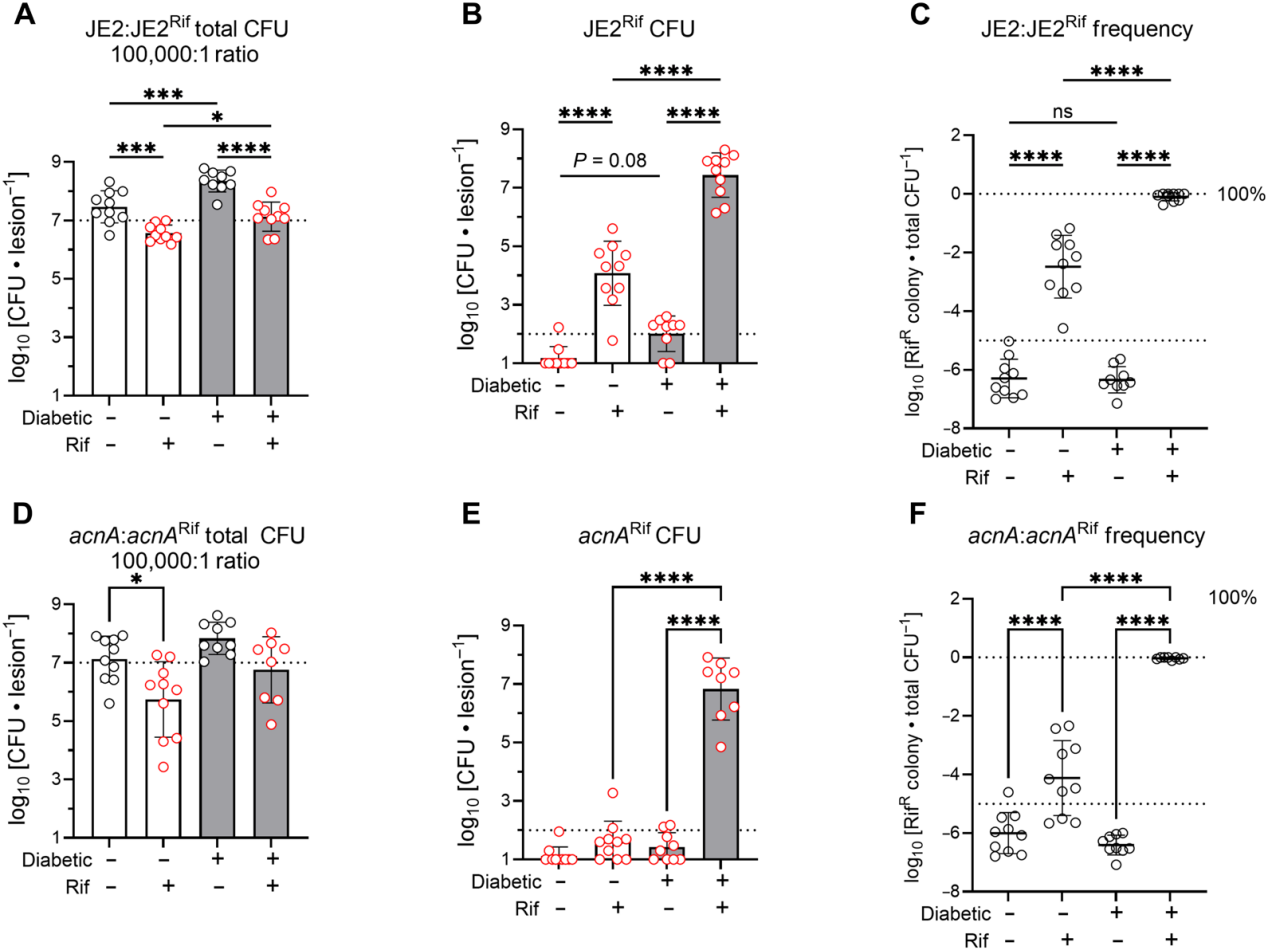
One hundred percent of *S. aureus* in the diabetic lesion become resistant to rifampicin following rifampicin treatment. Nondiabetic and diabetic mice were infected with a ratio of 100,000:1 WT:Rif^R^ *S. aureus* (**A** to **C**) or 100,000:1 *acnA*::Tn:*acnA*::Tn^Rif^ (**D** to **F**) and treated with or without rifampicin. (A) and (D) represent the total bacterial burden, (B) and (E) represent the enumerated Rif^R^ *S. aureus*, and (C) and (F) represent the endpoint resistance frequency. The dotted line in (A) and (D) denotes the 10^7^ CFU inoculum and that in (B) and (E) indicates the ~100 CFU Rif^R^ inoculum. Data are shown as the SD from the mean. *P < 0.05, ***P < 0.001, ****P < 0.0001, ns = not significant, ANOVA with Tukey’s correction for multiple comparisons.

To better understand how the diabetic environment contributes to the expansion of the Rif^R^ *S. aureus* population, we infected each mouse with a predetermined number of Rif^R^ CFUs (H481Y: MIC = 256 μg/ml). We isolated a Rif^R^ colony from a rifampicin-treated diabetic mouse lesion infected with *S. aureus* (WT JE2 strain) and designated it “D6R.” We verified an absence of major growth defects in vitro between D6R and the parent JE2 strain (fig. S5A). In addition, we verified that D6R does not have a competitive disadvantage in vitro (fig. S5B). Last, we confirmed that there is no competitive difference between D6R *S. aureus* and the parent strain in human macrophages (fig. S5C). We infected mice with 100 D6R CFUs and 10^7^ WT JE2 CFUs to assess the expansion of the Rif^R^ *S. aureus* population in our in vivo SSTI model after 5 days (Fig. 5, A to C). There was no difference in the level of competition between D6R and WT *S. aureus* in either diabetic or normal infection environments (fig. S6). Each diabetic mouse coinfected with WT and D6R *S. aureus* that was treated with rifampicin showed near 100% of the bacterial burden recovered from the lesion to be D6R *S. aureus*. This was a significantly higher amount (10^7^ CFUs versus 10^4^ CFUs, Fig. 5B) and at a higher frequency (~100% versus <1%, Fig. 5C) than Rif^R^ *S. aureus* recovered from the nondiabetic lesions of mice treated with rifampicin. These data show that the diabetic environment promotes the expansion of antibiotic-resistant mutants during antibiotic treatment. In addition, we observed a similar frequency of Rif^R^ *S. aureus* recovered from diabetic and nondiabetic animals in the absence of antibiotic pressure (Fig. 5C).

Having observed a role for *acnA* and potentially pyruvate flux through the TCA cycle in the emergence of Rif^R^ *S. aureus* (Fig. 4), we next sought to understand whether aconitase activity within the TCA cycle is essential for the propagation of resistance. We generated a transposon-insertion mutant of *acnA* in our isolated D6R JE2 strain, herein referred to as *acnA*::Tn^Rif^. We infected mice with a 100,000:1 ratio of *acnA*::Tn to *acnA*::Tn^Rif^ (Fig. 5, D to F). We again observed that each diabetic mouse treated with rifampicin showed near 100% of the bacterial burden recovered from the lesion to be Rif^R^ *S. aureus* (Fig. 5F). However, unlike with the WT D6R, we observed no significant proliferation of Rif^R^ *S. aureus* in nondiabetic mice (Fig. 5E). We concluded that aconitase activity is essential for expansion of Rif^R^ *S. aureus* during infection in nondiabetic mice, but dispensable for expansion in diabetic mice

### VISA growth is enhanced in diabetic infections

Vancomycin is one of the primary frontline antibiotics for treating bacteremia caused by methicillin-resistant *S. aureus* (MRSA) (*44*). The risk of infection with vancomycin intermediate-resistance *S. aureus* (VISA) is three times higher in patients with diabetes (*45*, *46*). VISA strains are derived from vancomycin-sensitive *S. aureus* (VSSA) that has acquired several mutations that alter cell wall synthesis, ultimately conferring intermediate resistance to vancomycin (*47*). The mutations associated with VISA result in growth defects caused by dysregulated cell wall synthesis, which is energetically taxing on the cell as many of the steps required for peptidoglycan synthesis require ATP (*48*–*51*). These mutations occur within the host and are selected for during vancomycin treatment in a similar manner to mutations conferring resistance to rifampicin (*52*). VISA isolates are less virulent and have slower growth rates compared to vancomycin-sensitive *S. aureus* (*44*, *53*). As such, we sought to determine whether a growth- and virulence-attenuated VISA strain could cause invasive infection within a diabetic infection.

**Fig. 6.**
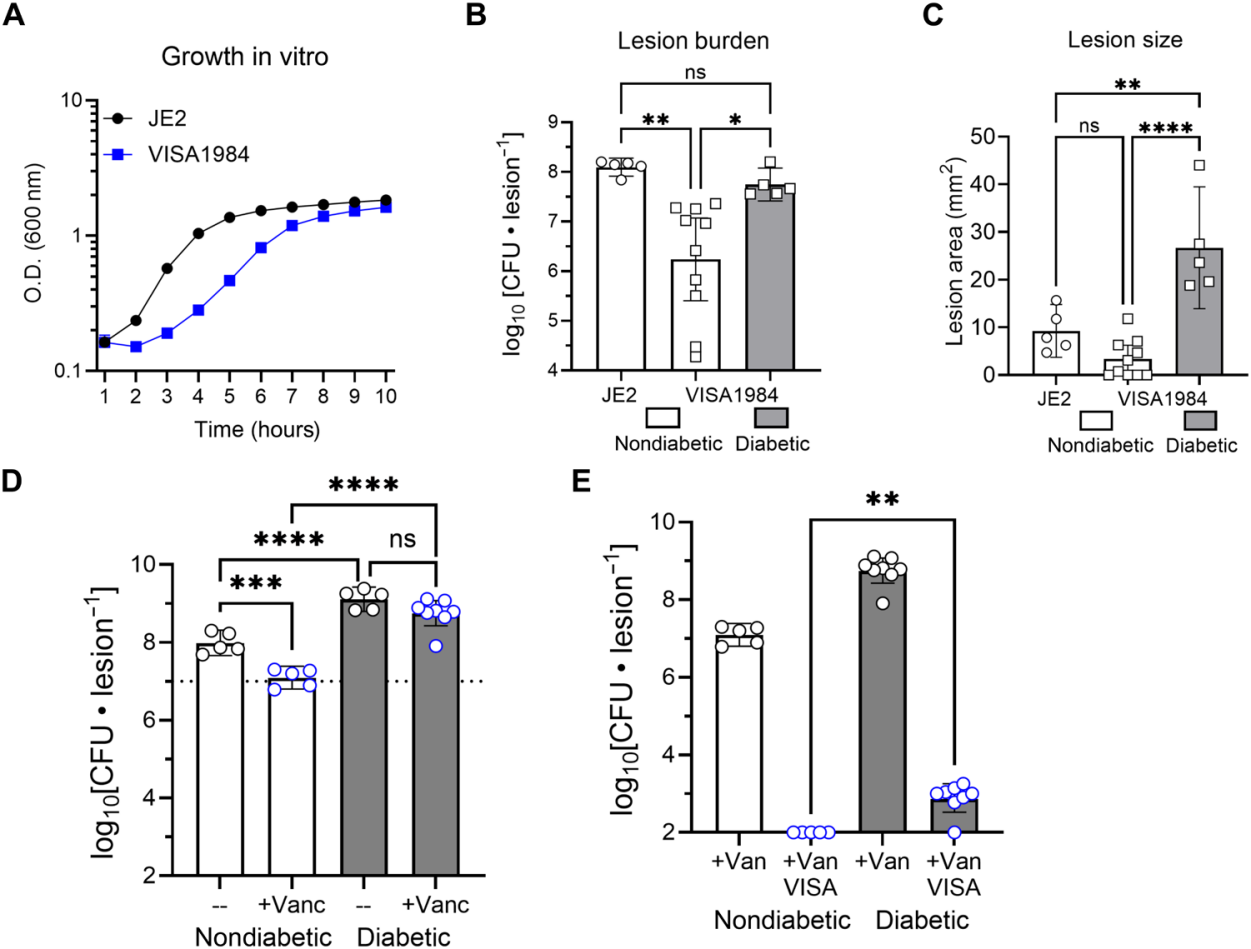
A clinical VISA strain (VISA1984) exhibits enhanced growth and virulence potential in diabetic mice and VISA is isolated from diabetic mice. (**A**) Growth of WT JE2 (black circles) and VISA 1984 (blue squares) was quantified in vitro. (**B**) Recovered CFUs of WT JE2 or VISA1984 from nondiabetic mice (white bars) or diabetic mice (gray bar). (**C**) Lesion sizes were quantified in nondiabetic infected mice (white bars) and diabetic infected mice (gray bar). (**D**) Total bacterial burden isolated from lesions on day 5 after daily vancomycin dosing. (**E**) Total bacterial burden isolated from vancomycin-treated mice and when plated onto media containing vancomycin (3 μg/ml). Data are shown as geometric mean with 95% CI. For (B) to (D), *P < 0.05, **P < 0.01, ***P < 0.001, ****P < 0.0001, ns = not significant, ANOVA with Tukey’s correction for multiple comparisons. For (E), **P = 0.0085, Mann-Whitney test.

Because diabetic immune suppression and hyperglycemia enhance *S. aureus* growth potential and virulence (*14*), we postulated that infection in diabetic animals would exacerbate VISA infection. We first confirmed that a characterized VISA, herein referred to as VISA1984, with an MIC of 6 μg/ml and isolated from a clinical infection (*54*) had slower growth compared to WT JE2 (Fig. 6A). We then infected mice with VISA1984 and assessed how well it could infect nondiabetic mice and diabetic mice. We observed that VISA1984 growth was extremely attenuated in nondiabetic mice relative to diabetic mice (Fig. 6B). In addition, VISA1984 formed significantly larger lesions in diabetic mice compared to nondiabetic mice. VISA1984 formed lesions that were even larger than the lesions formed by JE2 *S. aureus*, which is known to have a relatively high virulence potential (*55*–*57*) (Fig. 6C). We concluded that the diabetic infection microenvironment potentiates the proliferation and virulence of clinical VISAs. Because the diabetic environment restored the virulence potential of a clinically defined VISA, we wanted to assess whether we could isolate VISA from our diabetic model. We infected mice identically as shown in Fig. 1A. We dosed with 110 mg/kg vancomycin daily. We then plated the vancomycin-treated mouse samples onto plates containing vancomycin (3 μg/ml), which is a clinical breakpoint definition of VISA according to the European Committee on Antimicrobial Susceptibility Testing (Fig. 6E) (*58*). We only observed growth above our limit of detection in vancomycin-treated diabetic samples. These data provide strong evidence that the diabetic environment restores the virulence potential of clinical VISA and supports their evolution during infection.

### Insulin treatment reduces the emergence of antibiotic-resistant *S. aureus*

**Fig. 7.**
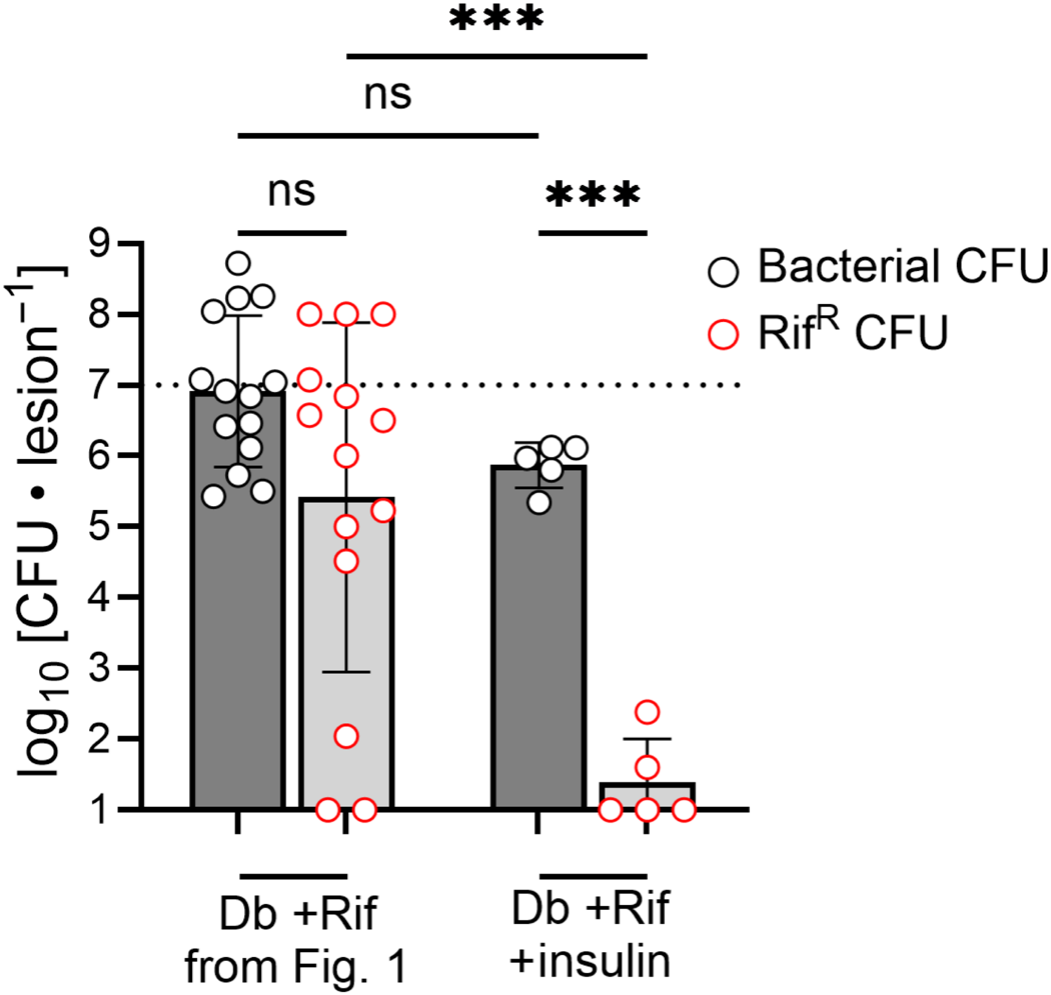
Insulin treatment controls the propagation of Rif^R^ *S. aureus*. Diabetic mice were treated with insulin daily and then infected with WT JE2. Next, mice were treated with rifampicin, and tissues were homogenized for bacterial burden quantification (white circles) and resistance quantification (red circles) (right-hand bars). Data on the left side of the graph are taken from Fig. 1 for comparison. Data are shown as the SD from the mean. ***P < 0.001, ns = not significant, ANOVA with Tukey’s correction for multiple comparisons.

To control disease, people with T1DM and insulin-dependent T2DM are prescribed insulin to manage hyperglycemia by maintaining functional insulin signaling. Uncontrolled DM (HbA1c ≥ 9) is associated with a higher risk for contracting severe bacterial infections compared to individuals without DM and individuals with controlled DM (*59*, *60*). Our STZ-treated mouse model induces diabetes by ablating insulin-producing pancreatic β cells of mice. To model control of hyperglycemia via insulin treatment in T1DM and insulin-dependent T2DM, we administered insulin daily to the diabetic mice, and used blood sugar as a measurement of diabetic control. Our treatment schedule was able to significantly reduce the non–fasting blood sugar of diabetic mice (fig. S3). Administering insulin to diabetic mice greatly reduced the emergence of Rif^R^ *S. aureus* (Fig. 7), despite only partially restoring normal blood glucose levels. These data indicate that controlling diabetes with insulin helps to prevent the emergence of antibiotic-resistant *S. aureus.*

## DISCUSSION

Concomitant to the increased incidence of DM worldwide is the emergence and expanding population of AMR bacterial pathogens (*1*, *2*). Poorly managed DM results in numerous systemic complications that directly contribute to the development of chronic bacterial infections, including hyperglycemia, myopathy, neuropathy, and impaired immune responses (*4*, *7*, *8*). The combined effects of impaired immune cell function with elevated blood and tissue glucose concentrations serve as an optimal niche for several bacterial pathogens, including *S. aureus* (*45*). As such, individuals with diabetes rely on conventional antibiotics to effectively clear infections, which inadvertently corresponds to increased rates of infection with MDR bacterial species (*28*, *31*, *61*, *62*). Despite sufficient evidence existing to suggest that the diabetic infection environment is a potential reservoir for antibiotic-resistant bacterial pathogens, current literature does not explore this phenomenon. Here, we demonstrate that the diabetic infection microenvironment can promote the emergence and proliferation of *S. aureus* with de novo antibiotic resistance in as little as 5 days.

Our combined data suggest that the diabetic microenvironment leads to the emergence of AMR, not through excess mutations (Fig. 1D and fig. S2), but through a heightened level of expansion potential of emergent antibiotic-resistant bacteria (Fig. 5). Although the diabetic environment may not increase the frequency of mutations in the bacterial population, the increased proliferation of *S. aureus* in diabetic tissues (Fig. 1B) likely supplements the potential of *S. aureus* to acquire mutations conferring resistance to antibiotics (fig. S1), which can, in turn, expand rapidly in a diabetic mouse under antibiotic pressure (Fig. 5). We observed spontaneous emergence of rifampicin-resistant *S. aureus* colonies in vitro at a rate of 1 in 10^7^ to 1 in 10^8^ CFUs (fig. S8). Considering our infection inoculum of 10^7^ CFUs, it is likely that a rifampicin-resistant mutant was able to emerge. However, our data suggest that only in a diabetic environment under antibiotic pressure can this Rif^R^ mutant thrive and expand.

Recent work shows that immune suppression can lead to the emergence of ciprofloxacin-resistant *Acinetobacter baumannii* using a sequential lung infection model in neutropenic mice (*32*). Here, we attempted to replicate diabetes-associated dysfunction of neutrophils and other phagocytes in our mouse model using rapamycin treatment to inhibit phagocyte bactericidal activity (*14*, *23*). Following rapamycin treatment, 50% of mice displayed infection with *S. aureus* that had developed de novo rifampicin resistance (Fig. 3B). In contrast, we did not detect the emergence of any Rif^R^ *S. aureus* in nondiabetic mice (Figs. 1C and 3B). Combined, these data suggest that the immune system plays a role within our model for preventing the emergence of AMR *S. aureus*. Notably, the AMR emergence in *A. baumannii* in immunocompromised mice was attributed to hypermutators in the population, whereas we did not observe any evidence of hypermutator *S. aureus* in the present study. Instead, our results suggest that Rif^R^ mutants are more likely to occur in diabetic mice as a function of the larger infecting population. We find that once a resistant mutant is present, it rapidly takes over in diabetic mice, but remains repressed in nondiabetic mice, and this rapid takeover appears to be primarily due to the increased glucose availability in the diabetic infection.

We were interested in determining what metabolic pathways were driving the rapid expansion of Rif^R^ *S. aureus* in diabetic mice. Pyruvate flux through the TCA cycle may play a role in AMR emergence, as dysfunction of *acnA* resulted in Rif^R^ being detected in only 50% of infected mice (Fig. 4, B and C). However, when rifampicin-resistant *acnA* mutants were introduced in the inoculum, they displayed no defect in their capacity to expand in diabetic mice under antibiotic pressure, suggesting the TCA cycle is not essential to the rapid expansion of resistant mutants observed in diabetic mice. One likely explanation is that the high level of glucose availability in the diabetic environment allows Rif^R^ *S. aureus* to use glycolysis for expansion in a process that does not require flux through the TCA cycle (*63*, *64*). In a hyperglycemic environment, *S. aureus* concomitantly reduces its synthesis of TCA cycle enzymes and up-regulates the expression of enzymes involved with glycolytic metabolism (*65*). We have previously shown that high-glucose environments can sensitize otherwise tolerant *S. aureus* to rifampicin treatment even when TCA cycle flux is incapacitated (*66*). Nevertheless, the current lack of glycolytic *S. aureus* mutants without substantial virulence defects makes further study of this metabolic pathway in our SSTI model difficult, as glycolysis is required for establishment of *S. aureus* infection (*11*). We attempted to subcutaneously infect diabetic mice with 10^7^ CFUs of a glycolytic mutant *S. aureus* strain with a deletion in *pfkA* (*11*), but we were unable to recover any *S. aureus* after 5 days of infection (fig. S9). In future studies, the direct examination of the role of overflow metabolism in the emergence of antibiotic resistance using mutants in pta, ackA, and ldh 1 and 2 will be interesting and may shed further light on the relationship between glucose availability and the expansion of resistant populations.

Although rifampicin is not recommended for clinical use as a monotherapy due to its well-described and readily observed resistance acquisition by *S. aureus*, it is, however, a powerful tool to study the emergence of AMR (*34*, *67*). Our study also made use of *S. aureus* with intermediate resistance to vancomycin, an antibiotic commonly used in clinical treatment of SSTIs, including DFI (*68*–*71*). We showed that a VISA clinical isolate was more fit and more virulent during infection of diabetic mice (Fig. 6). Shockingly, the VISA strain induced a lesion size in diabetic mice that was significantly larger than the lesion size formed by WT JE2 in nondiabetic mice (Fig. 6C). These results align with longitudinal clinical data from patients receiving long-term vancomycin treatment, demonstrating that clonal VISA isolates from the same patient exhibit an increased tolerance to vancomycin (*72*, *73*). The increased fitness and virulence of the VISA strain in the diabetic environment underscores the impact that diabetes can have on the emergence of AMR in *S. aureus* by demonstrating that an antibiotic-resistant *S. aureus* strain with attenuated growth exhibits enhanced growth and virulence in a diabetic infection.

We showed that even moderately controlling diabetes with insulin use significantly reduced Rif^R^ emergence (Fig. 7). This suggests that the emergence of AMR in the context of diabetes may only result when insulin signaling is sufficiently impaired and that even moderate control of diabetes may curb potential AMR emergence. Insulin treatment may potentially control AMR through restoring immune function, which is highlighted by previous studies showing that treatment with insulin at doses that do not resolve hyperglycemia still resolves immune cell dysfunction (*74*). In summary, our findings provide strong evidence to suggest that the diabetic infection environment is a potent reservoir for both the emergence and propagation of AMR bacteria, which is a profound threat to global health care systems given that the prevalence of diabetes and AMR are both rapidly increasing worldwide.

## MATERIALS AND METHODS

### Bacterial strains and growth conditions

The bacterial strains listed in Table 1 were used for in vitro and in vivo studies in this manuscript. *S. aureus* was grown in TSB (Remel) or TSB without dextrose (TSB −Glc; Bacto). Dextrose (15 mM; Thermo Fisher Scientific) or casamino acids (0.09% w/v; Bacto) were supplemented into TSB −Glc for fig. S4. Cultures were grown at 37°C shaking at 220 revolutions per minute (rpm). CFUs were enumerated by serial dilution on tryptic soy agar (TSA; Remel) for bacterial counts, or TSA supplemented with rifampicin (1 μg/ml; Thermo Fisher Scientific) for Rif^R^ *S. aureus* enumeration. Plates were incubated overnight at 37°C. Mutant bacterial strains were generated using previously described Φ11 phage transduction (*75*). PCR primers for mutant confirmation are listed in Table 2.

**Table 1.** Strain list.

Strain name	Genotype	Citation
*S. aureus* JE2	wild type (rifampicin sensitive 0.03 μg/ml)	(*75*)
*S. aureus* ∆G4	*glcA*::Kan^25^, *glcB*::erm^5^, *glcC*::Spec^100^, *glcU*::tet^10^	(*39*)
*S. aureus acnA*::Tn	*acnA*::Tn (erm)	(*75*)
*S. aureus* ∆*pfkA*	∆*pfkA* (clean deletion)	(*79*)
*S. aureus* D6R1	H481Y (rifampicin resistant 256 μg/ml)	(This study)
*S. aureus* VISA1984	SA1984 vancomycin intermediate-resistance *S. aureus*	(*54*)
*S. aureus acnA*::TnD6R1	H481Y, *acnA*::Tn	(This study)
*S. aureus 1902*::Tn	*pgl*::Tn (erm)	(*80*)

**Table 2.** Primer list.

Primer name	Sequence 5′-3′
Tn check 1	CTCGATTCTATTAACAAGGG
Tn check 2	GCTTTTTCTAAATGTTTTTTAAGTAAATCAAGTAC
*rpoB*-F1	GGCAGGTCAAGTTGTCCAATATG
*rpoB*-R1	TTAATCAGTAACTTCTTTTTGTGTTTCAGG
*rpoB*-seqF1	GCTGAGCCAATTGTAAATACTG
*rpoB*-seqR2	CCATATCGTAAAGTTGATTTAGATACAC
*acnA*::Tn Tn check	GAGCAAGGTATTACTAAAGTTTCC
*glcA* Tn check	GAGTTAAATTAAGCTGTGATGG
*glcB* Tn check	GCAGGCATGAGCAAACAAC
*glcC* Tn check	CTCAAAAGCTATATTGAGAATAATTAGG
*glcU* Tn check	GGGCAGAATGCTTTACAATAAC
*sodM* Tn check	GTGCTGCTATTGCGCGTTTAATTAC
∆*sodA* check F	GTAGTAGAATTTAAGCAAATTCTTTGTTG
∆*sodA* check R	GGTCTCATTTAAGAGACCGAAC
∆*pfkA* check F	CAGTTATAGAAAGGTATGTCGTCATG
∆*pfkA* check R	GGTCCAATTGTACATACAATTTTAG
*1902*::Tn check F	TTAAAATATGACACATACACCTTCAG

### Animal studies

In vivo experiments were carried out at the University of North Carolina at Chapel Hill under an Institutional Animal Care and Use Committee (IACUC)-approved protocol in an AAALAC-accredited facility. Seven-week old C57BL/6 mice were obtained from the Jackson Laboratory. Mice were checked daily during infection studies. Mice were rendered diabetic as described in Thurlow *et al.* (*14*)*.* Briefly, STZ (Sigma-Aldrich, CAS# 18883-66-4) was administered by intraperitoneal (i.p.) injection at 225 mg/kg 3 days before infection. On the day of infection, blood glucose levels were measured by tail-nick using a glucometer. Mice with blood glucose ≥300 mg/dl were considered hyperglycemic and mice with blood glucose below 300 mg/dl were excluded from studies. Mice were then subcutaneously infected with 1 × 10^7^ CFUs/ml bacteria. After 24 hours, mice were administered rifampicin at 25 mg/kg i.p. once daily for treatment infections. For Fig. 6D, mice were administered vancomycin at 110 mg/kg i.p. once daily for treatment. On Day 5, lesions were isolated and homogenized in 1 ml of PBS for bacterial burden and rifampicin resistance quantification. Resistance frequency was calculated by dividing the total rifampicin-resistant bacterial burden by the total bacterial population. For enumeration of bacterial resistant isolates, samples were plated onto TSA containing either rifampicin (1 μg/ml) for rifampicin-resistant mutants or TSA containing vancomycin (3 μg/ml) for VISA.

Rapamycin (Rapa) used in experiments was obtained from LC Laboratories (Woburn, MA). Mice were treated with Rapa (8 mg/kg) daily via the intraperitoneal route for 5 days and then rested for 2 days without treatment before subcutaneous infection with 10^7^ CFUs *S. aureus* as described above. Daily treatment with Rapa (8 mg/kg) resumed on the day of infection and persisted throughout the rest of the study period.

For experiments using insulin, mice were monitored daily for blood glucose after diabetes induction with STZ. At blood glucose level ≥ 300 mg/dl, mice were considered diabetic, and subcutaneously administered 6 U/day of insulin (Humulin R, Eli Lilly). If mice continued to have daily blood glucose readings ≥400 mg/dl, the daily dose of insulin was increased in 2-U increments to a maximum of 12 U/day. Diabetic mice with blood glucose 200–300 mg/dl were given a maintenance dose of 2 U/day insulin.

### Whole population genome sequencing

Whole population sequencing was done for three replicates per treatment. Populations from day 5 postinfection were sequenced, as well as the ancestral clone. The mouse-derived populations were plated in a lawn on TSA, cell density of approximately 1 × 10^12^ CFUs/ml, and a lawn scrape was taken for extraction and sequencing. DNA was extracted using the DNeasy blood and tissue kit (Qiagen). Extracted DNA was frozen and sent to SeqCoast Genomics (https://seqcoast.com) for 2 × 150-bp paired-end reads whole genome sequencing using an Illumina NextSeq2000 sequencing platform. Samples were prepped using the Illumina DNA Prep tagmentation kit and spiked with 1 to 2% PhiX control. Populations were sequenced at an average of 1.59 × 10^9^ ± 1.91 × 10^8^ bases per sample, resulting in an average depth of coverage of 550×.

Resulting paired-end reads were quality filtered and trimmed using Trimmomatic (v0.39) (criteria: LEADING:20 TRAILING:20 SLIDINGWINDOW:4:20 MINLEN:70) (*76*). Breseq (v0.36.1) was used for variant calling with default criteria (*77*). *S. aureus* JE2 genome (GenBank number GCF_002085525) was used as the reference genome. The Gdtools utility program was then used to remove mutations present in the reference ancestor genome from the mutations present in the evolved populations and annotate the resulting mutation output file (*77*). Plotting was done in R (v4.3.1) (www.r-project.org) with ggplot2 (v3.4.3) (*78*).

### In vitro studies

*S. aureus* and respective mutants were grown overnight in TSB −Glc at 37°C shaking at 225 rpm. The overnight cultures were back diluted 1:3000 in either TSB −Glc, TSB +Glc (15 mM glucose), or TSB +aa (0.09% w/v casamino acids), then grown to stationary phase at 37°C shaking at 225 rpm. At each time point, 10 μl of culture was removed and serially diluted for bacterial enumeration on TSA and resistance to rifampicin on TSA + rifampicin (1 μg/ml).

Growth curves were performed using a BioTek Plate Reader. Overnight cultures were diluted 1:100 in 200-μl wells filled with TSB.

### Human monocyte isolation

Blood was drawn from healthy volunteers into EDTA-coated phlebotomy tubes. An equal volume of 3% dextran T500 in 0.9% saline was added to sediment red blood cells. The top layer was removed and centrifuged at 250*g* for 10 minutes. The pellet was resuspended in 0.9% saline and layered over 10 ml of Ficoll-Paque plus before centrifugation at 400*g* for 40 min. The layer containing peripheral blood mononuclear cells (PBMCs) was removed, diluted 1:2 with PBS and washed twice with PBS. The PBMCs were plated onto low-adherence six-well tissue culture plates in RPMI 1640 (Gibco) + 10% fetal bovine serum (FBS) and l-glutamine (RPMI complete), macrophage colony-stimulating factor (M-CSF, 50 ng/ml), and penicillin-streptomycin. The macrophages were differentiated for 6 days before transferring to a 96-well tissue culture–treated plate at 5 × 10^4^ cells/well in RPMI complete. All volunteers gave informed consent for these studies.

*S. aureus* cultures were grown overnight in TSB 37°C with shaking at 225 rpm. The human monocyte-derived macrophages were infected by the addition of bacteria at a multiplicity of infection of 10. Phagocytosis was synchronized by centrifugation at 5 min at 500*g* before incubation for 30 min. The medium was removed and replaced with RPMI/10% FBS containing gentamicin (50 μg/ml) to kill extracellular bacteria. The macrophages were harvested after 18 hours by washing three times with 200 μl of PBS and lyses with 0.1% Triton X-100 in PBS.

### Statistical analysis

Statistical analysis was performed in GraphPad Prism v10. CFU and resistance frequency data were log transformed for analysis. Unless otherwise stated in the figure legends, comparisons between groups were performed using Student’s *t* test for two groups, and analysis of variance (ANOVA) with appropriate posttest for three or more groups.
