# Supplementary material for: Diabetes potentiates the emergence and expansion of antibiotic resistance
Source: Sci Adv. 2025 Feb 12;11(7):eads1591. doi: 10.1126/sciadv.ads1591 (PMC11817934; doi:10.1126/sciadv.ads1591)
Supplement: Supplementary file 1 — Figs. S1 to S7 [file sciadv.ads1591_sm.pdf]

Science Advances
AAAS

Supplementary Materials for

Diabetes potentiates the emergence and expansion of antibiotic resistance

John C. Shook *et al.*

Corresponding author: Lance R. Thurlow, thurlow@email.unc.edu; Brian P. Conlon, Brian_conlon@med.unc.edu

Sci. Adv. **11**, eads1591 (2025)
DOI: 10.1126/sciadv.ads1591

This PDF file includes:

Figs. S1 to S7

SUPPLEMENTAL FIGURES:

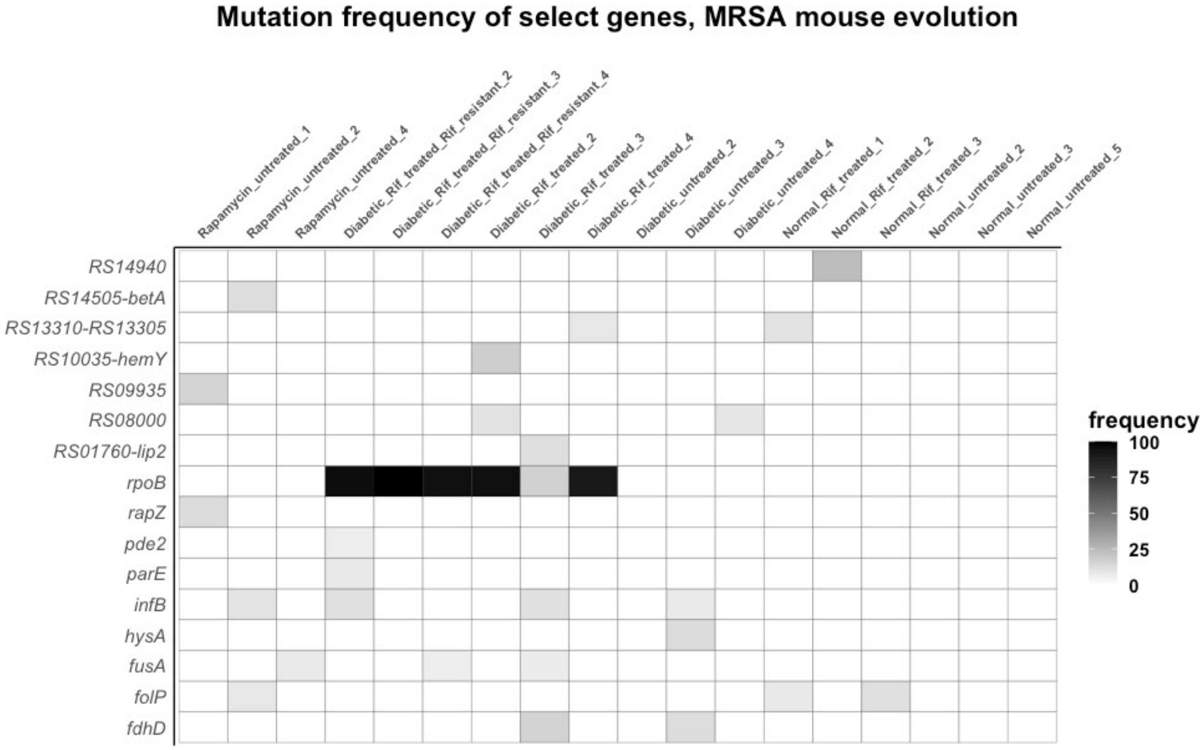

Figure S1.

Mutations of interest in all populations. In addition to identifying resistance mutations, we were also looking for *S. aureus* adaptation to the SSTI. All identified mutations with a frequency greater than 10% and/or found in more than one population.

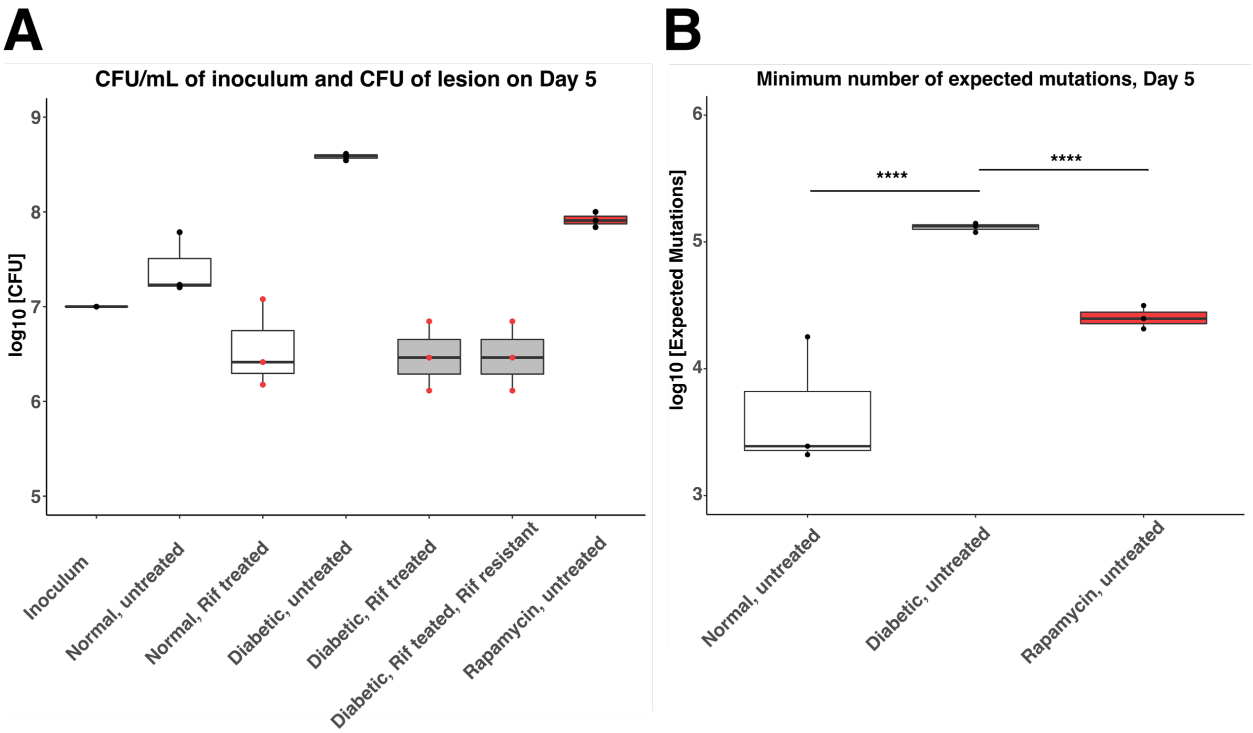

Figure S2

Total number of CFU per lesion recovered on TSA plates or TSA plates with rifampicin (denoted by 'Rif resistant') (A). Data in (A) incorporates data that are replotted from Fig. 1B and Fig 3A. Estimate of the minimum number of expected mutations based on initial and final population sizes and a constant mutation rate of 0.00035 mutations per cell division (B). Significant differences in mutations determined by ANOVA with Tukey's test for multiple comparisons, ****$p < 0.0001$.

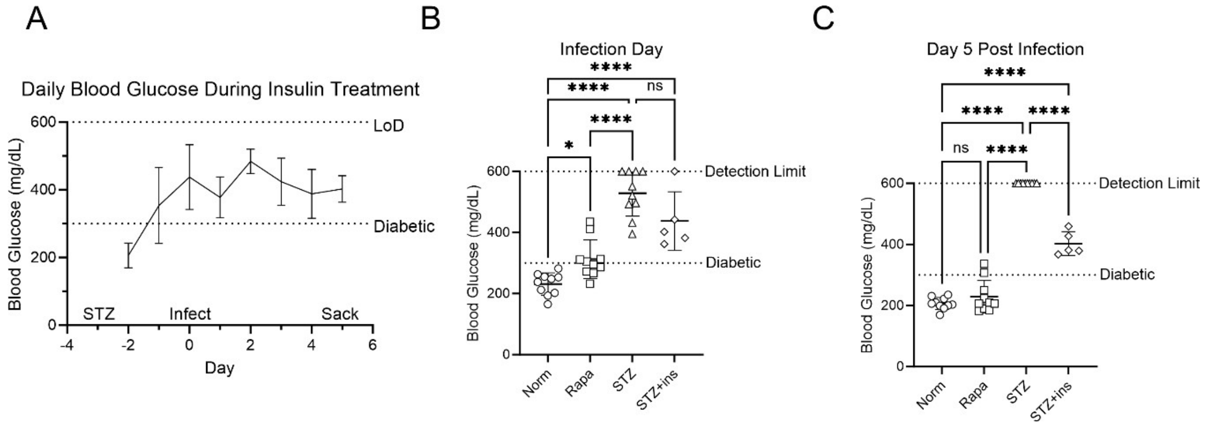

Figure S3.

Mouse blood glucose levels during study period. (A) Blood glucose levels from mice treated with streptozotocin (STZ), taken daily before subcutaneous injection of insulin. Insulin administration was started on the day STZ-treated mice became diabetic. Mice were considered diabetic when their blood glucose reading > 300 mg/mL. Blood glucose levels were compared on **(B)** the day of *S. aureus* infection and **(C)** five days later between groups: untreated, non-diabetic mice, rapamycin-treated mice (Rapa), STZ-treated mice (STZ), and STZ-treated mice treated daily with insulin (STZ+ins). Data is represented as mean+/-SD. **p* < 0.05, *****p* < 0.0001, ns = not significant, Tukey's test for multiple comparisons.

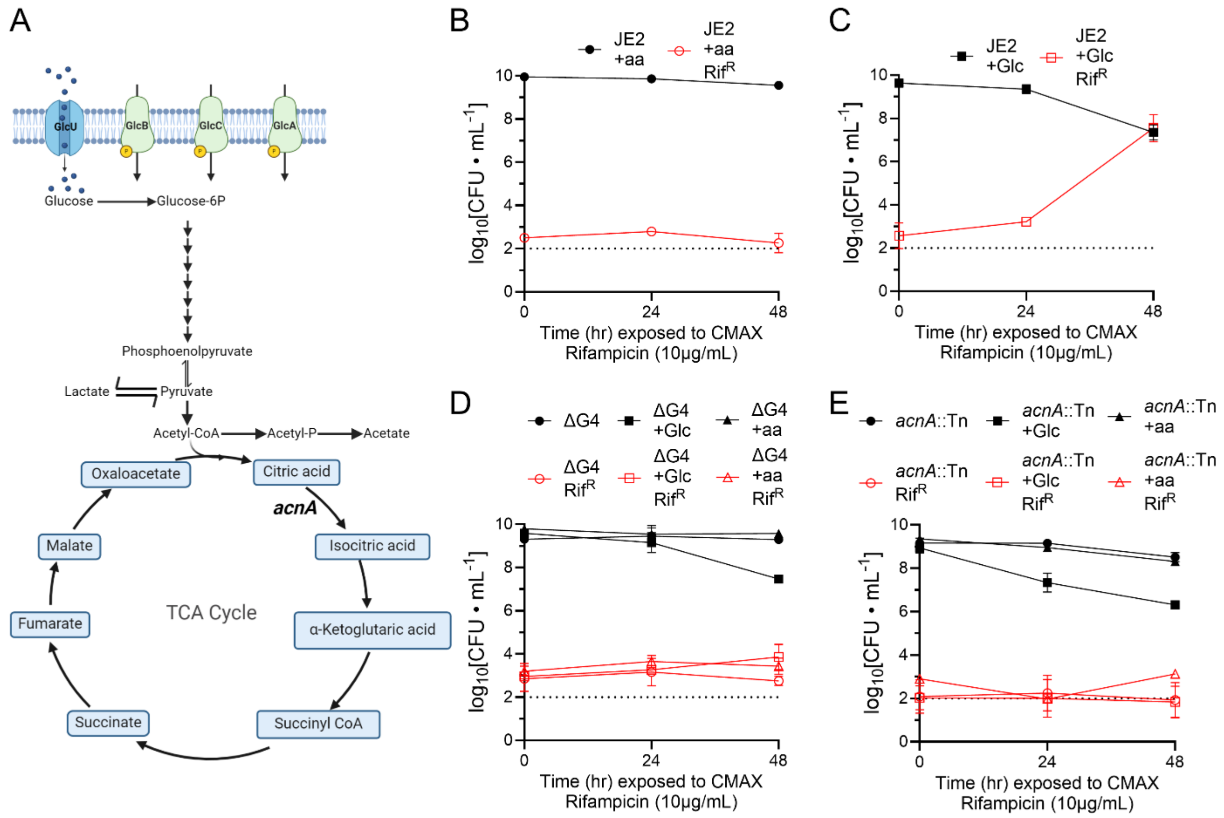

Figure S4:

Expansion of Rif^R *S. aureus* is dependent on glycolytic and TCA cycle metabolism *in vitro*. *S. aureus* was grown to stationary phase and then assessed for bacterial burden and Rif^R burden at time = 0, time = 24hr, and time = 48 hours. The approximate maximum concentration of a drug achieved in human serum (CMAX) rifampicin (10µg/mL) was added at time = 0. Bacterial burdens were assessed by plating on TSA (black solid shapes) or TSA with 1µg/mL rifampicin (red open shapes) **(A)** Generalized depiction of the carbon metabolism of *S. aureus*. **(B)** WT *S. aureus* supplemented with carbon equivalent casamino acids at t=0 and t=24. **(C)** WT *S. aureus* supplemented with 15mM glucose at t=0 and t=24. **(D)** The quadruple glucose transporter knockout, ΔG4. **(E)** The TCA cycle mutant, *acnA*::Tn.

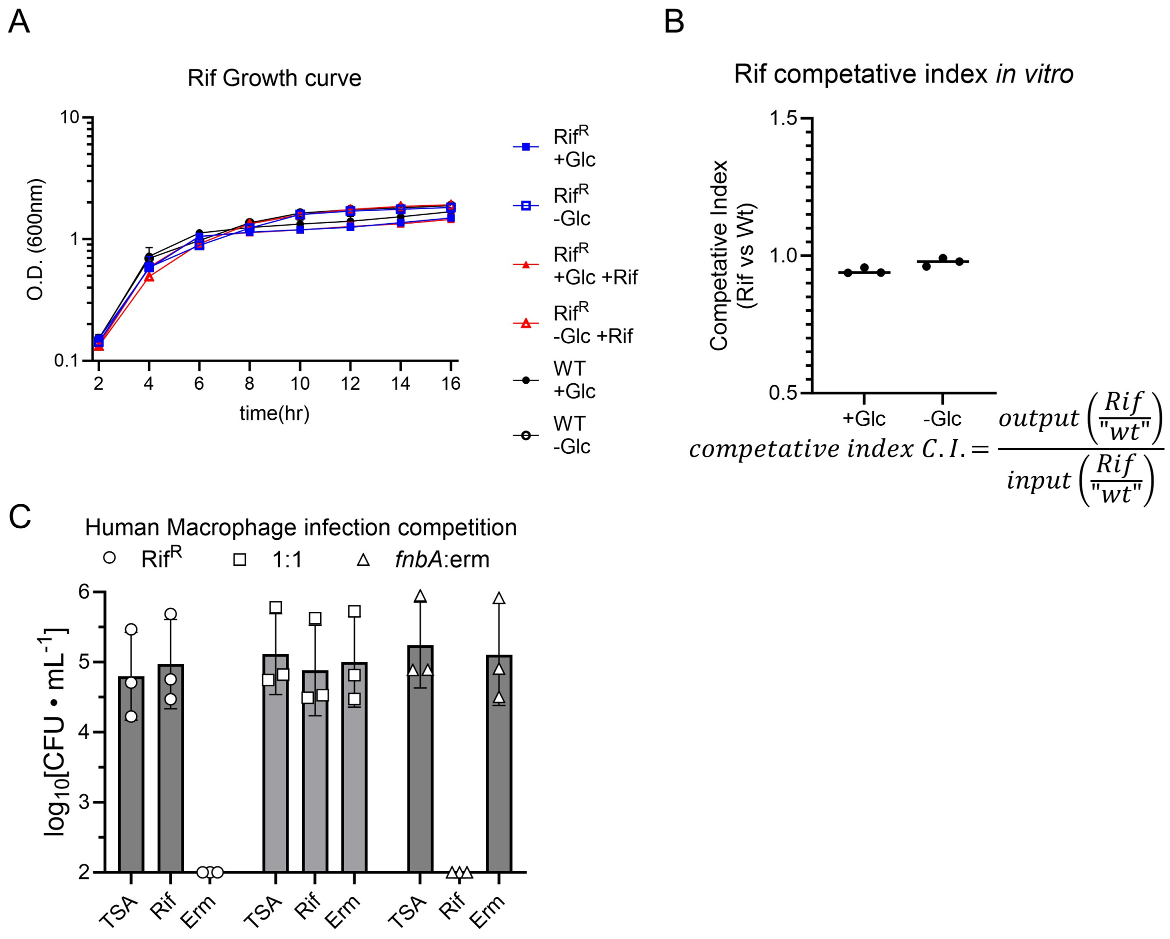

Figure S5.

D6R is not less fit than WT *S. aureus in vitro*. The D6R mutant was characterized for any growth defects. **(A)** Overnight cultures of WT JE2 and D6R (Rif^R) were diluted 1:100 into TSA +/- Glucose. Black circles = JE2, Blue Squares = D6R, red triangles = D6R supplemented with rifampicin. **(B)** Overnight bacterial cultures of an *fnbA*::Tn mutant and D6R were diluted to an OD_{600} = 1.0 and then mixed 1:1 in TSA +/- Glucose. Bacteria were plated onto plates containing erythromycin (erm) or rifampicin (rif) before and after incubation, and CFU were enumerated. Competitive index was calculated by dividing the output by the input. **(C)** Human macrophages were infected with a 1:1 mixture of the *fnbA*::Tn mutant and D6R at an MOIof 10. Bacterial CFU were quantified on TSA, or plates containing rifampicin or erythromycin.

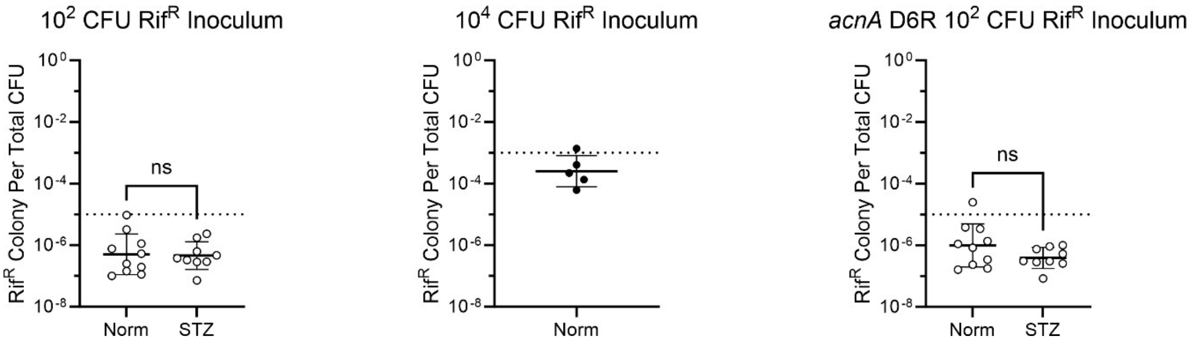

Figure S6.

Rifampicin-resistant D6R *S. aureus* strain is outcompeted by rifampicin-sensitive parent JE2 strain in SSTI. (A) 10^2 CFU D6R and 10^7 CFU parent JE2 strain were inoculated subcutaneously into non-diabetic mice and diabetic mice (STZ) and were allowed to compete in the infection environment for 5 days without the presence of antibiotics. For each mouse, the enumerated ratio between rifampicin-resistant and rifampicin-sensitive CFU recovered from the lesion was calculated to represent the competition result between the D6R and parent JE2 strains. The theoretical amount of rifampicin-resistant JE2 inoculated into each mouse was <1 CFU. **(B)** Competition results from inoculum containing 10^4 CFU D6R and 10^7 CFU parent JE2 strain in untreated non-diabetic mice. Dotted line represents the expected ratio of D6R CFU : JE2 CFU if there was no competition. Data represented as mean +/- SD for each group. ns = not significant by *t*-test.

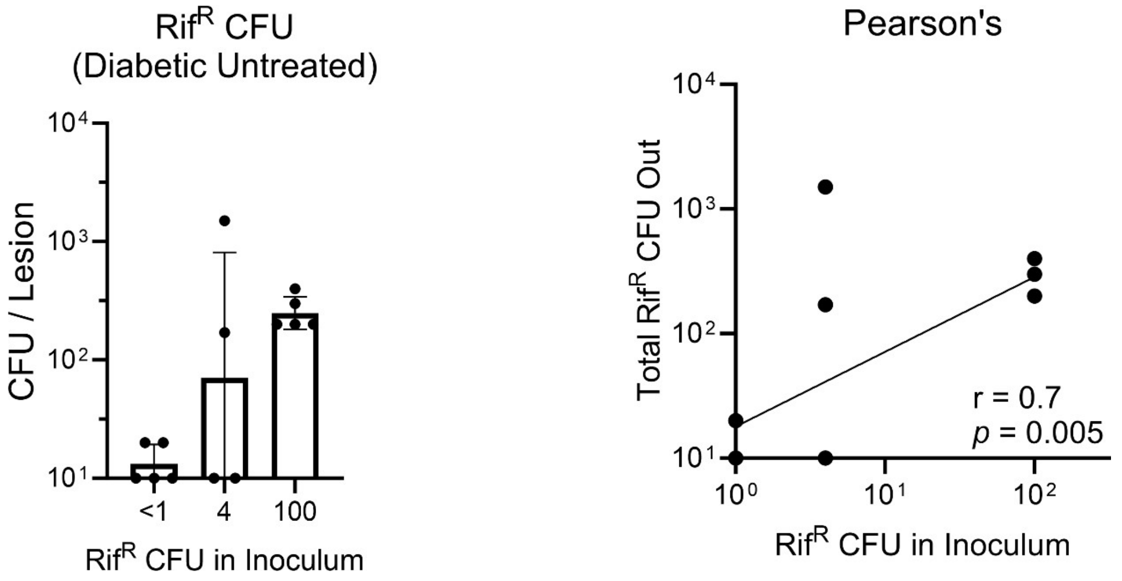

Figure S7.

The number of rifampicin-resistant *S. aureus* CFU isolated from untreated diabetic SSTI correlates the number of rifampicin-resistant *S. aureus* CFU enumerated in the inoculum. (A) Rifampicin-resistant *S. aureus* (Rif^R) present in the inoculum were enumerated as the theoretical number of Rif^R CFU contained in each subcutaneous inoculation with 10^7 WT *S. aureus.* Five days after inoculation in diabetic mice without antibiotic treatment, Rif^R CFU were enumerated from the resulting lesions. Data is represented as mean+/-SD. **(B)** Pearson's correlation analysis comparing the number of Rif^R CFU theoretically inoculated in each mouse with the number of Rif^R CFU recovered from the resulting lesion 5 days later.
